# Variability in breath alcohol concentration profiles during controlled social drinking

**DOI:** 10.1093/jat/bkag038

**Published:** 2026-06-03

**Authors:** Ryan S O’Connor, Jennifer Wang, Jay R Vargas

**Affiliations:** School of Criminal Justice and Criminalistics, California State University Los Angeles, 5151 State University Drive, Los Angeles, CA 90032, United States; School of Criminal Justice and Criminalistics, California State University Los Angeles, 5151 State University Drive, Los Angeles, CA 90032, United States; School of Criminal Justice and Criminalistics, California State University Los Angeles, 5151 State University Drive, Los Angeles, CA 90032, United States

## Abstract

Controlled alcohol studies often use standardized dosing conditions to characterize absorption and elimination. The present study evaluated breath alcohol concentration (BrAC) profiles under supervised social drinking conditions that permitted variability in beverage consumption and food intake. Forty-two adult participants consumed self-selected 80-proof mixed drinks with ad libitum access to food. Drinking duration ranged from 45 to 172 min (mean 108 min, median 110 min), reflecting substantial variability in consumption time across participants. BrAC was measured at approximately 30-min intervals using an Intox EC/IR II until return to zero. Peak BrAC ranged from 0.040 to 0.117 g/210 L (mean 0.087). Time from the final drink to peak ranged from −23 to 76 min (mean 17 min, median 13 min). The mean elimination rate was 0.0186 g/210 L/hr (SD 0.0027, range 0.0134 to 0.0261). Most participants reached peak concentration shortly after drinking ceased, although delayed peaks of more than 1 hr were observed in some individuals. Representative curves demonstrated variability in absorption timing, including occasional multi-peak profiles, while post-peak elimination was generally linear. These findings characterize the range of BrAC–time patterns observed under supervised social drinking conditions and provide context for interpreting alcohol measurements in applied settings.

## Introduction

Alcohol pharmacokinetics has long been described using mathematical relationships proposed by Erik M. P. Widmark (1889–1945) in the early twentieth century [1, 2]. These relationships describe the distribution of ethanol as a function of dose, body characteristics, and an assumed elimination rate. The Widmark framework continues to inform forensic practice, where it is applied in the interpretation of breath and blood alcohol results and in estimating earlier alcohol concentrations in driving investigations [3, 4].

Many applications of these models rely on assumptions regarding absorption and elimination, including the presence of a single peak concentration and a relatively linear post-absorptive decline [5]. Controlled laboratory studies that support these assumptions typically employ fixed dosing, standardized timing, and restricted food intake. Under these conditions, ethanol concentration-time profiles often demonstrate a consistent absorption phase followed by a well-defined elimination phase [6].

Outside of controlled settings, alcohol consumption occurs under more variable, real-world conditions. Factors such as drinking pace [7], beverage composition [8, 9], recent food intake [10, 11], type of food [12, 13], gastric emptying variability [14], carbonated mixers [15], medications [16, 17], underlying health conditions [18, 19], and intersubject physiological differences [20, 21] can influence both the rate and extent of ethanol absorption. These factors may contribute to concentration-time profiles that differ in timing, shape, and reproducibility compared to those observed under controlled conditions [22, 23].

The present study was designed to characterize pharmacokinetic variability under conditions that more closely reflect typical drinking behavior. Participants consumed self-selected mixed drinks containing 80 proof spirits while eating freely and drinking at their preferred pace. Breath alcohol concentrations were measured repeatedly using an evidentiary-grade instrument. The primary aim was to quantify variability in peak concentration, time to peak, elimination rate, and overall curve characteristics. A secondary aim was to describe the range of concentration-time profiles observed under these conditions relative to commonly applied pharmacokinetic assumptions.

## Methods

### Participants

Forty-two adults volunteered for controlled social drinking sessions held at California State University Los Angeles. Most participants were university students or family members. A licensed physician conducted a medical screening to rule out chronic medical conditions or contraindicated medications. Written informed consent was obtained from all participants. All study procedures were reviewed and approved by the Institutional Review Board of California State University Los Angeles. Each participant received a $20 gift card at the completion of the session. Individuals remained at the study site until their breath alcohol concentration returned to 0.000 g/210 L.

### Study environment

All sessions were carried out indoors under direct supervision. Participants consumed alcoholic beverages in a social setting while study staff monitored safety and collected breath samples. Individuals were permitted to stop drinking at any point. Alcoholic beverages were prepared and provided by study personnel using measured volumes of 80 proof spirits. Participants were allowed to select mixers and adjust beverage composition to preference. Study staff recorded the amount of alcohol dispensed and monitored consumption throughout the session. Additional drinks were provided based on the dosing plan and real-time breath alcohol measurements.

### Alcohol dose estimation

Dose estimates were calculated using a Widmark style formulation adapted to breath alcohol units to approximate the number of drinks required to achieve a target peak BrAC of approximately 0.10 g/210 L within 60 min.

The equation used was:


$$n=\frac{[BAC_{\text{target}}+(0.025\cdot H)]\cdot W\cdot V_{d}}{5.0\cdot A}$$

In this equation, *n* represents the number of standard drinks, defined as 1.5 oz (44 mL) of 80 proof spirits, corresponding to approximately 14 g of ethanol. The target BrAC was 0.10 g/210 L. An elimination rate of 0.025 g/210 L per hour was used as a practical input parameter for dose estimation. This value represents the upper end of commonly reported elimination rates and was selected to reduce the likelihood of underestimating the amount of alcohol required to approach the target concentration. The term *H* represents hours since drinking began, *W* represents body weight in pounds, and *V_d_* is an individually estimated volume of distribution calculated using anthropometric variables, including body weight and height, based on sex-specific regression equations described by Seidl *et al.* [24]. Sex-specific parameters were applied for each participant based on reported sex at the time of dosing. The constant 5.0 converts fluid ounces of ethanol to grams (1 fl oz ≈ 23.3 g) and scales the Widmark formulation to approximate breath alcohol concentration units expressed as g/210 L. The term *A* represents the ethanol content of one standard drink in fluid ounces.

Based on these estimates, individualized drink plans were developed for each participant. Initial dosing typically consisted of a single (1.5 oz) or double (3.0 oz) drink, followed by additional fractional doses (0.75 oz) as needed. Breath alcohol measurements obtained during the session were used to guide further dosing, and additional drinks were administered when participants had not yet approached the target concentration. Participants consumed beverages at their preferred pace within this guided framework.

Accordingly, the total amount of ethanol consumed varied across participants. Participants consumed a mean of 4.9 standard drinks (SD 1.3, range 2.5–8.0), corresponding to approximately 69 g of ethanol on average.

### Breath alcohol collection

Breath alcohol measurements were obtained using an Intox EC/IR II instrument (Intoximeters Inc., St. Louis, MO, USA), which incorporates electrochemical fuel cell detection for quantitative breath alcohol measurement together with infrared monitoring used for breath profile and sample quality control functions. The instrument was provided, maintained, and calibrated by the Los Angeles Police Department. Standard breath alcohol testing procedures were followed, including observation prior to breath sampling to minimize the potential influence of residual mouth alcohol.

Baseline measurements were obtained prior to alcohol consumption to confirm zero breath alcohol concentration. Subsequent measurements were collected at approximately 30-minute intervals throughout the session until BrAC returned to zero.

Each analytical sequence consisted of a system suitability check using a standard gas (SYST), followed by a blank measurement (0.000 g/210 L), a subject breath sample, a second blank, and a replicate subject breath sample obtained within a few minutes. Only results reported by the instrument as “test status: success” were included in the analysis. When replicate measurements were obtained at a given time point, the mean value was used for subsequent pharmacokinetic calculations.

### Data processing and pharmacokinetic measures

Times were converted to minutes relative to the recorded start of drinking. Peak breath alcohol concentration (Cmax) and time to peak (Tmax) were extracted for each participant. In addition to Tmax calculated from the start of drinking, the time from the final drink to peak breath alcohol concentration was calculated and reported as Tmax (last drink). Elimination rates were determined by fitting a linear regression model to the post-peak portion of each curve and converting the slope to an hourly rate expressed in g/210 L/hr. Curve shapes were assessed for features such as multiple peaks or irregularities in the elimination phase. All data processing, pharmacokinetic calculations, and statistical summaries were performed using custom Python scripts developed with standard scientific computing libraries, including pandas, NumPy, and SciPy, with figures generated using Matplotlib.

## Results

### Participant characteristics

Forty-two participants completed the study. Demographic variables were not available for all participants due to incomplete data collection during some sessions; however, all 42 participants were included in pharmacokinetic analyses based on available breath alcohol data, and sex and anthropometric measurements required for dose estimation were recorded for all participants.

Sample sizes for each variable are reported in Table 1. Participant body weights ranged from 96–274 pounds (43.5–124.3 kg), and estimated body mass index values ranged from approximately 18–48 kg/m^2^.

**Table 1:** Participant demographics.

Variable	Mean	SD	Minimum	Maximum	*N*
Age (years)	28.3	7.0	22	43	37
Height (inches)	65.6	3.5	59	75	39
Weight (pounds)	167.0	43.3	96	274	39
Weight (kg)	75.8	19.6	43.5	124.3	39
Body mass index (kg/m²)	27.3	6.0	18.2	48.1	39
Sex (M/F)	16/26	—	—	—	42

Summary statistics for age, height, body weight, and calculated body mass index (BMI) for study participants. Values are reported as mean, standard deviation (SD), minimum, maximum, and sample size (*N*).

### Drinking duration

**Figure 1:**
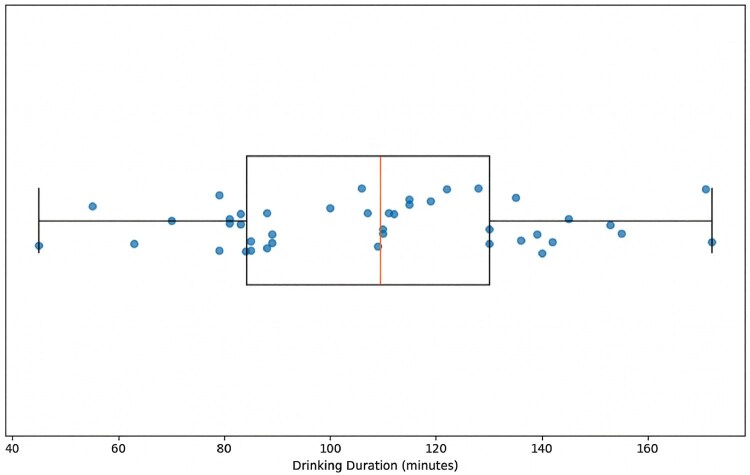
Distribution of drinking duration among participants. Box and scatter plot showing drinking duration, defined as the elapsed time in minutes from the recorded start of drinking to the recorded end of drinking for each participant.

Drinking duration varied substantially across participants, ranging from 45 to 172 min, with a mean of 108 min and a median of 110 min. The interquartile range extended from 84 to 130 min. Figure 1 illustrates the distribution of drinking duration, demonstrating considerable variability in beverage consumption time under the study conditions.

### Peak breath alcohol concentrations

**Figure 2:**
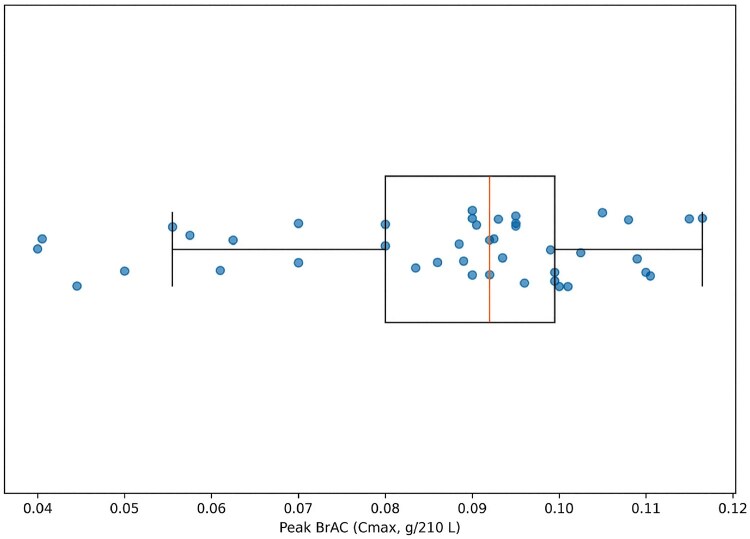
Distribution of peak breath alcohol concentrations (Cmax). Box and scatter plot showing peak BrAC values (g/210 L) across all participants. Points represent individual observations and the box indicates the interquartile range with median.

Peak breath alcohol concentrations ranged from 0.040 to 0.117 g/210 L. The mean peak breath alcohol concentration Cmax was 0.087 g/210 L. Figure 2 shows the distribution of peak values across participants. Some participants reached or exceeded the target concentration of approximately 0.10 g/210 L, while others remained below this level. Despite individualized dosing based on anthropometric measures and Widmark based calculations, substantial variability in observed peak breath alcohol concentrations was present. Summary pharmacokinetic parameters are presented in Table 2.

**Table 2:** Summary of pharmacokinetic measures.

Parameter	Mean	SD	Min	Max	*N*
Peak BrAC, Cmax (g/210 L)	0.0866	0.0203	0.0400	0.117	42
Tmax from last drink (min)	17.1	23.7	−23	76	42
Elimination rate from breath (g/210 L/hr)	0.0186	0.0027	0.0134	0.0261	42

Peak breath alcohol concentration (Cmax), time from the final drink to peak concentration (Tmax last drink), and elimination rate (β). Values are reported as mean, standard deviation (SD), minimum, maximum, and sample size (*N*). Elimination rates were estimated from linear regression of the post-peak portion of each concentration–time curve.

### Time to peak concentration

Time from the final drink to peak breath alcohol concentration varied across participants. Tmax (last drink) ranged from −23 to 76 min, with a median value of 13 min and a mean of 17 min (SD 23.7 min). Negative values indicate that peak breath alcohol concentration occurred before the participant finished drinking. Most participants reached peak concentrations shortly after drinking ceased, although some individuals exhibited delayed peaks occurring more than one hour after the final drink. Because drinking duration varied across participants, time to peak referenced to the start of drinking showed greater dispersion and is less informative for interpretation under these study conditions.

### Elimination rates

**Figure 3:**
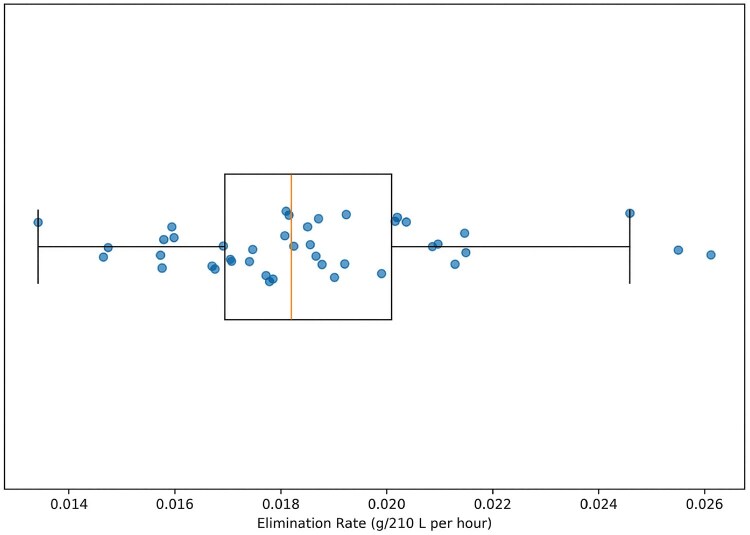
Distribution of alcohol elimination rates. Box and scatter plot showing elimination rates (g/210 L/hr) estimated from the post-peak portion of each participant’s concentration–time curve.

Estimated elimination rates showed a mean value of 0.0186 g/210 L/hr with a standard deviation of 0.0027 g/210 L/hr. Individual elimination rates ranged from 0.0134 to 0.0261 g/210 L/hr. Figure 3 displays the distribution of elimination rates across participants. Many sessions produced highly linear post peak descending limbs, while others showed modest departures from linearity or irregular descending patterns.

### Representative concentration time curves

**Figure 4:**
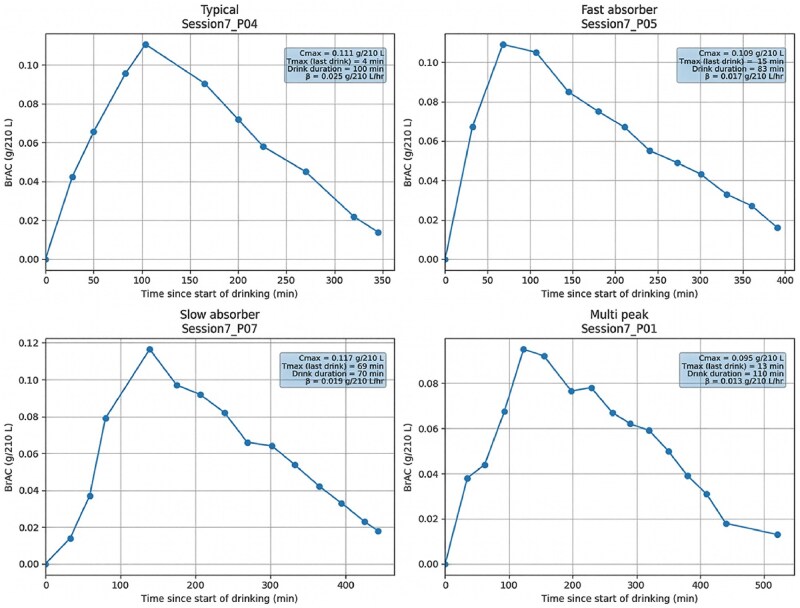
Representative real-world drinking curves obtained during controlled social-drinking sessions. Each panel shows BrAC (g/210 L) versus time (minutes from drinking start) for a single participant. Panels illustrate a typical single-peak profile, relatively rapid absorption, delayed peak concentration, and a multi-peak profile. For comparability, single-peak exemplars were selected from participants with broadly similar drinking durations. Annotated values include peak concentration (Cmax), time to peak relative to the final drink, drinking duration, and elimination rate (β). All curves originate from a confirmed baseline BrAC of 0.000 g/210 L prior to alcohol consumption, which is displayed at time zero.

Figure 4 presents four representative concentration–time curves selected to illustrate characteristic patterns observed in the dataset. The panels include a typical single-peak profile with a well-defined post-peak decline, a participant with relatively rapid absorption and early peak concentration, a participant with delayed time to peak, and a multi-peak profile with more than one local maximum within the concentration–time profile. Differences in the rate of rise to peak and the slope of the post-peak decline are evident across panels. Together, these examples illustrate the range of concentration–time profiles observed across sessions under the study conditions.

## Discussion

This study demonstrates that alcohol pharmacokinetics during social drinking exhibit considerable variability, even under controlled conditions with individualized dosing. Breath alcohol concentrations were used as the primary measurement in this study. It is recognized that BrAC may underestimate venous blood alcohol concentrations depending on the assumed blood-to-breath partition ratio, particularly during the post-absorptive phase. Peak BrAC, time to peak, and overall curve shape varied widely despite Widmark-style dose calculations. Time to peak showed substantial variability, ranging from −23 to 76 min relative to the final drink, whereas values referenced to drinking onset were more widely dispersed and less informative under variable drinking durations. Most participants reached peak breath alcohol concentrations shortly after drinking ceased, with a median lag of approximately 13 min and three quarters of participants peaking within roughly 30 min. However, delayed peaks of more than one hour after the final drink were also observed. These findings indicate that although many individuals may enter the post-absorptive phase within the first hour after drinking ends, the transition from absorption to elimination cannot be assumed to occur at a fixed time point for all individuals.

Multi-peak profiles and delayed peaks illustrate how real-world drinking behaviors can produce concentration–time patterns that differ from the simplified single-peak models often described in classical pharmacokinetic studies. These observations illustrate that the timing of the transition between absorption and elimination can vary across individuals, which may affect how fixed time thresholds are applied in forensic interpretations.

Elimination rates in the present study (mean 0.0186 g/210 L/h) remained within the range commonly reported in forensic literature and were generally characterized by linear post-peak slopes. Once the post-absorptive phase was established, elimination behavior was consistent with values typically applied in forensic back-calculation. Modest curvature or stair-step patterns were observed in some descending limbs, likely reflecting physiological variability, although continued absorption cannot be entirely excluded; however, these features emphasize the importance of careful interval selection when estimating elimination rates.

Overall, the naturalistic design of this study captures absorption and elimination behaviors that are commonly encountered in real-world drinking. These findings support the continued use of Widmark-based approaches in appropriate circumstances while emphasizing that assumptions regarding the timing of absorption completion should be applied cautiously and evaluated in the context of the available case-specific information.

## Conclusion

Real-world breath alcohol concentration time profiles frequently differ from the idealized patterns described in classical pharmacokinetic models. In this supervised social drinking study, peak concentrations, time to peak, elimination rates, and curve shapes varied widely despite individualized dose calculations based on Widmark-style equations. Delayed peaks, multi-peak absorption patterns, and modest irregularities in elimination were commonly observed. While elimination rates were generally consistent with values commonly applied in forensic practice and post peak elimination was often linear, the timing of peak concentration and the transition from absorption to the post absorptive phase were not uniform across participants. These findings suggest that although many individuals may reach peak concentration shortly after drinking ends, the timing of absorption completion varied across individuals and was not consistently aligned to a single time point. The results, therefore, are broadly consistent with the continued application of Widmark-based approaches under appropriate conditions. Overall, these data provide an empirical description of the variability encountered in supervised social drinking conditions and may assist in contextual interpretation of breath alcohol measurements.

## References

[1] Widmark E. Die Theoretischen Grundlagen Und Die Praktische Verwendbarkeit Der Gerichtlich-Medizinischen Alkoholbestimmung. Urban-Schwarzenberg, 1932.

[2] Andreasson R , Jones AW. The life and work of Erik M. P. Widmark. Am J Forensic Med Pathol 1996;17:177–90. 10.1097/00000433-199609000-00001 8870865

[3] Jones AW. Evidence-based survey of the elimination rates of ethanol from blood with applications in forensic casework. Forensic Sci Int 2010;200:1–20. 10.1016/j.forsciint.2010.02.021 20304569

[4] Jones AW. Pharmacokinetics of ethanol—issues of forensic importance. Forensic Sci Rev 2011;23:91–136. 26231237

[5] Reed TE. The myth of "the average alcohol response". Alcohol 1985;2:515–9. 10.1016/0741-8329(85)90126-0 4026973

[6] Holford NH. Clinical pharmacokinetics of ethanol. Clin Pharmacokinet 1987;13:273–92. 10.2165/00003088-198713050-00001 3319346

[7] Dubowski KM. Absorption, distribution and elimination of alcohol: highway safety aspects. J Stud Alcohol Suppl 1985;10:98–108. 10.15288/jsas.1985.s10.98 3862865

[8] Roine RP , Gentry RT, Lim RT Jr. et al Comparison of blood alcohol concentrations after beer and whiskey. Alcohol Clin Exp Res 1993;17:709–11. 10.1111/j.1530-0277.1993.tb00824.x 8333604

[9] Mitchell MC Jr. , Teigen EL, Ramchandani VA. Absorption and peak blood alcohol concentration after drinking beer, wine, or spirits. Alcohol Clin Exp Res 2014;38:1200–4. 10.1111/acer.12355 24655007 PMC4112772

[10] Lin Y , Weidler DJ, Garg DC et al Effects of solid food on blood levels of alcohol in man. Res Commun Chem Pathol Pharmacol 1976;13:713–22. 1265346

[11] Singh BN. Effects of food on clinical pharmacokinetics. Clin Pharmacokinet 1999;37:213–55. 10.2165/00003088-199937030-00003 10511919

[12] Pikaar NA , Wedel M, Hermus RJ. Influence of several factors on blood alcohol concentrations after drinking alcohol. Alcohol Alcohol 1988;23:289–97. 3166629

[13] Jones AW , Jonsson KA, Kechagias S. Effect of high-fat, high-protein, and high-carbohydrate meals on the pharmacokinetics of a small dose of ethanol. Br J Clin Pharmacol 1997;44:521–6. 10.1046/j.1365-2125.1997.t01-1-00620.x 9431825 PMC2042884

[14] Oneta CM , Simanowski UA, Martinez M et al First pass metabolism of ethanol is strikingly influenced by the speed of gastric emptying. Gut 1998;43:612–9. 10.1136/gut.43.5.612 9824340 PMC1727307

[15] Roberts C , Robinson SP. Alcohol concentration and carbonation of drinks: the effect on blood alcohol levels. J Forensic Leg Med 2007;14:398–405. 10.1016/j.jflm.2006.12.010 17720590

[16] Edelbroek MA , Horowitz M, Wishart JM et al Effects of erythromycin on gastric emptying, alcohol absorption and small intestinal transit in normal subjects. J Nucl Med 1993;34:582–8. 8455074

[17] Amir I , Anwar N, Baraona E et al Ranitidine increases the bioavailability of imbibed alcohol by accelerating gastric emptying. Life Sci 1996;58:511–8. 10.1016/0024-3205(95)02316-x 8569424

[18] Acevedo MB , Eagon JC, Bartholow BD et al Sleeve gastrectomy surgery: when 2 alcoholic drinks are converted to 4. Surg Obes Relat Dis 2018;14:277–83. 10.1016/j.soard.2017.11.010 29305304 PMC5844810

[19] Booker JL , Renfroe K. The effects of gastroesophageal reflux disease on forensic breath alcohol testing. J Forensic Sci 2015;60:1516–22. 10.1111/1556-4029.12847 26110903

[20] Piekoszewski W , Gubala W. Inter- and intra-individual variability of ethanol pharmacokinetics over a long period of time. Pol J Pharmacol 2000;52:389–95. 11334232

[21] Friel PN , Baer JS, Logan BK. Variability of ethanol absorption and breath concentrations during a large-scale alcohol administration study. Alcohol Clin Exp Res 1995;19:1055–60. 10.1111/j.1530-0277.1995.tb00988.x 7485816

[22] Fraser AG , Rosalki SB, Gamble GD et al Inter-individual and intra-individual variability of ethanol concentration-time profiles: comparison of ethanol ingestion before or after an evening meal. Br J Clin Pharmacol 1995;40:387–92. 10.1111/j.1365-2125.1995.tb04561.x 8554941 PMC1365158

[23] Sedman AJ , Wilkinson PK, Sakmar E et al Food effects on absorption and metabolism of alcohol. J Stud Alcohol 1976;37:1197–214. 10.15288/jsa.1976.37.1197 979272

[24] Seidl S , Jensen U, Alt A. The calculation of blood ethanol concentrations in males and females. Int J Legal Med 2000;114:71–7. 10.1007/s004140000154 11197633

